# Real-World Outcomes of Frontline Multimodal Treatment Strategies for Localized Extranodal NK/T-Cell Lymphoma, Nasal Type: A Single-Center Vietnamese Cohort Study

**DOI:** 10.3390/curroncol33070435

**Published:** 2026-07-21

**Authors:** Huong Nguyen Thi Thu, Dang Nguyen Van, Yen Le Thi, Tung Nguyen Thanh, Manh Pham Duy, Nga Tran Thi Giang, Quang Le Van

**Affiliations:** 1 Department of Quan Su Medical Oncology, Vietnam National Cancer Hospital, Hanoi 100000, Vietnam; nguyenthu_huong@hmu.edu.vn (H.N.T.T.); leyenbvk@gmail.com (Y.L.T.); 2 Department of Oncology, Hanoi Medical University, Hanoi 100000, Vietnam; leonguyenthanhtung@gmail.com (T.N.T.); phamduymanh@hmu.edu.vn (M.P.D.);; 3 Department of Head and Neck Radiation Oncology, Vietnam National Cancer Hospital, Hanoi 100000, Vietnam; 4 Department of Hematologic Oncology, Vietnam National Cancer Hospital, Hanoi 100000, Vietnam; 5 Oncology Center, Thai Binh General Hospital, Thai Binh City 06106, Vietnam

**Keywords:** extranodal natural killer/T-cell lymphoma, nasal type (ENKTL-NT), radiotherapy, multimodal treatment, asparaginase, PINKE, survival outcomes

## Abstract

Introduction: Extranodal natural killer/T-cell lymphoma, nasal type (ENKTL-NT), is a rare and aggressive lymphoma for which real-world data from resource-limited settings remain scarce. This study evaluated the efficacy and safety of frontline multimodal treatment strategies for localized ENKTL-NT. Methods: We retrospectively analyzed 62 patients with localized ENKTL-NT treated at the Vietnam National Cancer Hospital between May 2019 and June 2025. Patients received concurrent chemoradiotherapy followed by VIPD or VIDL, or sequential chemoradiotherapy with GELOX/PGEMOX. Treatment responses, survival outcomes, and toxicities were assessed. Results: The median age was 44 years, and 67.7% of patients had stage I disease. Baseline EBV-DNA positivity was detected in 46.8%, while 54.8% had PINKE scores of 1–2. The overall response rate was 93.5%, including an 85.5% complete response rate. Grade 3–4 neutropenia was the most common adverse event (18.7%). The estimated 3-year progression-free survival (PFS) and overall survival (OS) rates were 76.3% and 79.5%, respectively. Multivariable Cox analysis identified PINKE risk stratification as an independent predictor of both PFS and OS. Conclusions: Frontline multimodal treatment strategies achieved high response rates, favorable survival outcomes, and manageable toxicities in localized ENKTL-NT. The PINKE score remained an important prognostic factor, supporting risk-adapted treatment selection in routine clinical practice.

## Simple Summary

Extranodal natural killer/T-cell lymphoma, nasal type (ENKTL-NT), is a rare and aggressive lymphoma that occurs more frequently in Asian populations. Although multimodal treatment combining radiotherapy and chemotherapy is currently recommended, real-world data from low- and middle-income countries remain limited. In this study, we evaluated 62 Vietnamese patients with localized ENKTL-NT treated with three frontline multimodal treatment strategies at a national cancer center. Overall, treatment outcomes were favorable, with high response rates, encouraging long-term survival, and manageable toxicities. Patients who achieved complete response experienced significantly better survival outcomes, while the PINKE prognostic model effectively identified patients at higher risk of disease progression and death. These findings reflect the ongoing evolution of routine clinical practice in Vietnam and underscore the feasibility of implementing contemporary, individualized treatment strategies despite the constraints of a resource-limited healthcare setting.

## 1. Introduction

Extranodal natural killer/T-cell lymphoma, nasal type (ENKTL-NT), is an aggressive subtype of non-Hodgkin lymphoma that arises predominantly from natural killer (NK) cells and, less commonly, cytotoxic T cells. It is closely associated with Epstein–Barr virus (EBV) [1,2]. ENKTL-NT usually arises in the nasal cavity or paranasal sinuses and commonly presents with nasal symptoms such as obstruction or epistaxis. Although ENKTL-NT is rare in Western countries, it represents a relatively common lymphoma subtype in Latin America and East Asia, including Vietnam. The disease occurs more frequently in men and is often diagnosed at a localized stage; nevertheless, clinical outcomes remain considerably poorer than those observed in most other lymphoma subtypes owing to its aggressive biological behavior, propensity for tissue necrosis, and high risk of relapse and systemic dissemination [2].

Historically, treatment approaches based solely on radiotherapy or anthracycline-containing chemotherapy regimens were associated with unsatisfactory clinical outcomes and substantial relapse rates [3]. The limited therapeutic efficacy of anthracyclines in ENKTL-NT is largely attributable to the overexpression of P-glycoprotein by NK/T lymphoma cells, resulting in intrinsic multidrug resistance. In contrast, regimens incorporating agents unaffected by P-glycoprotein-mediated efflux, particularly platinum compounds and asparaginase, have demonstrated significantly improved antitumor activity [4,5]. Consequently, contemporary treatment strategies increasingly favor combined chemoradiotherapy using non-anthracycline, asparaginase-based regimens, which have consistently yielded superior response rates and survival outcomes across multiple clinical studies [6,7,8]. Nevertheless, despite these therapeutic advances, an internationally standardized treatment paradigm has yet to be established, primarily owing to the rarity and biological heterogeneity of the disease, as well as the limited availability of large prospective randomized trials [8].

In Vietnam, previously published studies have predominantly evaluated individual treatment regimens, particularly the VIPD protocol, largely owing to the historical unavailability of L-asparaginase-based therapies. Consequently, comprehensive real-world analyses comparing multiple frontline therapeutic strategies remain limited [9]. In this context, the present study was conducted to evaluate the real-world outcomes of patients with localized extranodal NK/T-cell lymphoma, nasal type, treated at the Vietnam National Cancer Hospital using three distinct first-line regimens in accordance with National Comprehensive Cancer Network (NCCN) recommendations. Furthermore, data from low- and middle-income countries remain scarce, particularly in Southeast Asia, where treatment selection is frequently influenced by resource availability, reimbursement policies, and access to asparaginase-containing regimens. To our knowledge, this study represents the largest real-world Vietnamese cohort evaluating three frontline multimodal treatment strategies for localized ENKTL-NT and one of the few studies from Southeast Asia reporting long-term survival outcomes together with prognostic analyses. Consequently, real-world evidence from this setting is needed to better inform treatment decisions and optimize outcomes.

## 2. Materials and Methods

### 2.1. Patients

Eligible patients fulfilled the following inclusion criteria: histopathologically confirmed ENKTL-NT according to the 2016 World Health Organization (WHO) classification; stage IE–IIE disease based on the Lugano staging system; measurable lesions identified on PET/CT, magnetic resonance imaging (MRI), or computed tomography (CT) in accordance with the 2014 Lugano response criteria; age ≥ 16 years; no prior anti-lymphoma therapy; and receipt of first-line treatment consisting of concurrent chemoradiotherapy with weekly cisplatin with or without adjuvant chemotherapy, or sequential/alternating chemoradiotherapy. In addition, eligible patients had an ECOG performance status of 0–2 and adequate baseline hematologic, hepatic, and renal function. Laboratory eligibility criteria included a white blood cell count ≥ 4 G/L, platelet count ≥ 100 G/L, hemoglobin ≥ 100 g/L, serum AST/ALT ≤ 2 × ULN, total bilirubin ≤ 1.5 × ULN, and serum creatinine ≤ 1.5 × ULN. Patients were excluded if they had another malignancy, severe uncontrolled comorbidities, incomplete clinical records, discontinued treatment for non-medical reasons, or declined participation in the retrospective analysis.

### 2.2. Methods

This retrospective cohort study was conducted at K Hospital. We collected clinical data from consecutive patients with localized extranodal NK/T-cell lymphoma, nasal type (ENKTL-NT), who were diagnosed and treated between May 2019 and June 2025. Patients meeting the predefined inclusion and exclusion criteria were included in the study. The study protocol was reviewed and approved by the Ethics Committee of Hanoi Medical University (Approval No. 128/QĐ-ĐHYHN) on 17 January 2025. The study was conducted in accordance with the Declaration of Helsinki and its later amendments. The reporting of this study conforms to STROBE guidelines [10]. The STROBE checklist is provided as Supplementary File S1.

Clinical characteristics, laboratory findings, imaging results, treatment-related data, and follow-up evaluations were systematically collected from medical records and supplemented by retrospective clinical assessments. Data collection procedures and longitudinal follow-up were retrospectively standardized to ensure data consistency, reduce potential information bias, and maintain strict adherence to institutional treatment protocols. No patients were lost to follow-up during the study period.

The study endpoints were predefined prior to analysis. The primary endpoint was treatment response. Secondary endpoints included treatment modalities administered, overall survival (OS), progression-free survival (PFS), factors associated with treatment outcomes, hematologic toxicities, and non-hematologic adverse events.

To reduce selection bias, all consecutive eligible patients treated during the study period were included. Information bias was minimized through standardized data extraction from electronic medical records and predefined outcome definitions.

### 2.3. Treatment Protocol

Treatment strategies in the present study were categorized into three standardized multimodal therapeutic approaches for localized extranodal NK/T-cell lymphoma, nasal type [11]. The study cohort comprised three sequential treatment eras reflecting changes in routine clinical practice, drug availability, and institutional treatment protocols in Vietnam over time. Patients treated between 2019 and 2020 received concurrent chemoradiotherapy followed by the VIPD regimen (Arm 1). From 2020 to 2023, the institutional treatment strategy transitioned toward concurrent chemoradiotherapy followed by the VIDL regimen (Arm 2). Subsequently, between 2023 and 2025, the preferred frontline approach shifted to sequential GELOX/PGEMOX chemotherapy combined with radiotherapy (Arm 3), reflecting increasing adoption of asparaginase-based strategies in accordance with contemporary clinical evidence and evolving treatment availability in Vietnam.

For patients treated with concurrent chemoradiotherapy (CCRT), the treatment protocol consisted of involved-field radiotherapy combined with weekly radiosensitizing cisplatin, followed by adjuvant chemotherapy. In Arm 1, patients received radiotherapy to a total dose of 50 Gy together with weekly cisplatin at 30 mg/m^2^ for 5 weeks, followed by three cycles of the VIPD regimen. The VIPD regimen comprised etoposide 100 mg/m^2^, ifosfamide 1200 mg/m^2^, and cisplatin 33 mg/m^2^ administered on days 1–3, in combination with dexamethasone 40 mg on days 1–4. In Arm 2, patients underwent radiotherapy at a total dose of 40–44 Gy with concurrent weekly cisplatin for 4 weeks, followed by two cycles of the VIDL regimen. The VIDL protocol incorporated the same doses of etoposide, ifosfamide, and dexamethasone administered on days 1–3, while cisplatin was replaced by L-asparaginase at a dose of 4000 IU/m^2^ administered on alternate days between days 8 and 20.

Arm 3 employed a sequential “sandwich” chemoradiotherapy strategy consisting of two cycles of induction chemotherapy followed by radiotherapy and an additional two to four cycles of consolidation chemotherapy administered every 3 weeks. The chemotherapy backbone comprised gemcitabine 1000 mg/m^2^ and oxaliplatin 85 mg/m^2^ on day 1, combined with either PEG-asparaginase 2500 IU/m^2^ on day 1 or L-asparaginase 6000 IU/m^2^ administered from days 1 to 7. Radiotherapy was initiated within one week after completion of induction GELOX/PGEMOX chemotherapy and delivered to a total dose of 56 Gy.

All patients received involved-field radiotherapy using either IMRT or VMAT, delivered in daily 2-Gy fractions. Patients with extensive nasal–paranasal disease or multiple nodal sites were more likely to receive the sequential “sandwich” treatment strategy.

### 2.4. Monitoring and Evaluation Procedure

All patients underwent a rigorously standardized monitoring and evaluation protocol throughout the study period:

**Baseline evaluation:** Prior to treatment initiation, all patients underwent comprehensive clinical assessment, including detailed physical examination and fiberoptic nasopharyngoscopy to evaluate the extent of mucosal involvement. Baseline staging investigations comprised magnetic resonance imaging (MRI) of the head and neck in combination with either computed tomography (CT) of the chest, abdomen, and pelvis or ^18F-fluorodeoxyglucose positron emission tomography/computed tomography (^18F-FDG PET/CT) for systemic disease evaluation. Quantitative plasma Epstein–Barr virus DNA (EBV-DNA) levels were measured at baseline using real-time polymerase chain reaction (PCR).

**Interim assessment:** Clinical monitoring was performed throughout treatment, including weekly evaluations during radiotherapy and assessment at each chemotherapy cycle during systemic treatment. In Arms 1 and 2, interim radiologic evaluation using CT/MRI or PET/CT was conducted three weeks after completion of the concurrent chemoradiotherapy phase. In Arm 3, interim imaging assessment was performed following two cycles of induction GELOX/PGEMOX chemotherapy, approximately one week after completion of the second cycle. Serial plasma EBV-DNA measurements were performed in selected patients during treatment when considered clinically indicated but were not routinely obtained for all patients. Treatment decisions were based primarily on clinical assessment, endoscopic findings, and imaging evaluation rather than changes in EBV-DNA levels.

**Response assessment:** Treatment response was assessed four weeks after completion of first-line therapy using the 2014 Lugano response criteria. Complete response (CR) indicated the disappearance of all clinical, endoscopic, and radiologic evidence of disease. For the purpose of analysis, responders included patients who achieved either CR or partial response (PR), whereas non-responders included those with stable disease (SD) or progressive disease (PD).

**Long-term follow-up:** Patients were followed every three months during the first two years and every six months thereafter for up to five years. Follow-up included clinical examination, fiberoptic nasopharyngoscopy, imaging studies as clinically indicated, and plasma EBV-DNA testing when available. EBV-DNA was not measured routinely during treatment, and treatment decisions were based primarily on clinical assessment and imaging findings.

### 2.5. Statistical Analysis

All statistical analyses were performed using R software (version 4.6.0; R Foundation for Statistical Computing, Vienna, Austria). Baseline characteristics were summarized using descriptive statistics. Continuous variables were expressed as means with standard deviations or medians with ranges, whereas categorical variables were presented as counts and percentages. Comparisons between categorical variables were performed using Fisher’s exact test. OS and PFS were estimated using the Kaplan–Meier method and compared across subgroups using the log-rank test. Variables included in the multivariable Cox proportional hazards models were selected a priori based on clinical relevance, previously reported prognostic significance in ENKTL, and a directed acyclic graph (DAG)-informed causal framework. To minimize model overfitting given the limited number of events, only key prognostic variables were retained in the final multivariable models. Hazard ratios (HRs) and corresponding 95% confidence intervals (CIs) were reported. The completeness of all variables was assessed before statistical analysis. Variables with missing data were analyzed using a complete-case approach. No imputation procedures were performed because the proportion of missing data was low for all variables included in the survival analyses. All statistical tests were two-sided, and *p*-values < 0.05 were considered statistically significant.

### 2.6. Toxicity Assessment and Safety Monitoring

Adverse events (AEs) were recorded throughout treatment, either before each treatment cycle or whenever clinically significant symptoms occurred. Hematologic and non-hematologic toxicities were graded according to the National Cancer Institute Common Terminology Criteria for Adverse Events (NCI-CTCAE), version 5.0. 

**Clinical and laboratory monitoring:** Comprehensive clinical evaluations were performed before each chemotherapy cycle. Routine laboratory investigations included complete blood count parameters, comprising hemoglobin concentration, white blood cell count with differential, and platelet count, together with biochemical assessments including lactate dehydrogenase (LDH), uric acid, beta-2 microglobulin, blood urea nitrogen, serum creatinine, aspartate aminotransferase (AST), alanine aminotransferase (ALT), and total bilirubin levels.

**Asparaginase-related toxicity monitoring:** Patients receiving asparaginase-containing regimens were routinely monitored for pancreatic and coagulation abnormalities. Laboratory tests included serum amylase, lipase, fibrinogen, prothrombin time (PT), international normalized ratio (INR), and activated partial thromboplastin time (aPTT). These tests were obtained at baseline, before each treatment cycle, and every 1–2 weeks during therapy, with additional assessments performed when clinically indicated.

**Radiotherapy-related toxicity assessment:** Radiotherapy-related adverse events, including dermatitis, mucositis, and xerostomia, were assessed throughout the course of radiotherapy. Toxicity was graded using the National Cancer Institute Common Terminology Criteria for Adverse Events (NCI-CTCAE), version 5.0.

## 3. Results

### 3.1. Baseline Characteristics

A total of 62 eligible patients with localized ENKTL-NT were included in the study. Baseline characteristics are summarized in Table 1. The median age of the cohort was 44 years (range, 17–71 years), and most patients were male (75.8%). Stage IE disease accounted for 67.7% of cases, while 45.2% of patients had a PINKE score of 0. Significant differences in baseline characteristics were observed among treatment groups with respect to EBV-DNA status (*p* = 0.026), disease stage (*p* = 0.008), and PINKE score (*p* = 0.001). Patients treated with GELOX/PGEMOX more frequently presented with stage IIE disease, EBV-DNA levels ≥1000 copies/mL, and higher PINKE scores. No significant between-group differences were observed for age, sex, ECOG performance status, B symptoms, primary tumor site, LDH level, or β2-microglobulin concentration. Given the rarity of ENKTL, all consecutive eligible patients treated during the study period were included, which may partly explain the observed imbalance in baseline risk characteristics among treatment groups.

### 3.2. Treatment Adherence and Protocol Completion

Adherence to the predefined therapeutic strategy was consistently high across all treatment arms at our institution. Among the 43 patients treated with cisplatin-based concurrent chemoradiotherapy (CCRT), all successfully completed the radiotherapy component of treatment. Overall, 74.4% of patients received five weekly cisplatin cycles, whereas the remaining 25.6% completed four cycles, with a mean delivered radiation dose of 48.9 Gy.

Among 19 patients in Arm 3, 17 received GELOX, and 2 received PGEMOX; 94.7% of patients successfully completed the planned two induction chemotherapy cycles prior to radiotherapy, which was administered at a mean dose of 56 Gy. Only one patient (5.3%) received a single induction cycle because of early disease progression. Dose modifications during the concurrent treatment phase were uncommon. One patient in the CCRT cohort required a 25% chemotherapy dose reduction secondary to grade 2 nephrotoxicity, while one patient in the GELOX/PGEMOX cohort underwent a similar dose reduction because of grade 3 thrombocytopenia.

Transition to the adjuvant systemic treatment phase also demonstrated substantial treatment compliance, although minor variations were observed among regimens. Within the VIPD cohort (*n* = 27), 63.0% of patients completed all three planned adjuvant cycles, whereas 22.2% and 7.4% received two and one cycle, respectively; two patients (7.4%) did not proceed to adjuvant chemotherapy. In the VIDL cohort (*n* = 16), treatment adherence was particularly favorable, with 93.7% of patients completing both planned adjuvant cycles and only one patient (6.3%) receiving a single cycle. Among patients treated with sequential GELOX/PGEMOX chemoradiotherapy, 84.2% completed both planned post-radiotherapy consolidation cycles, 10.5% received one consolidation cycle, and one patient (5.3%) did not undergo post-radiotherapy chemotherapy. During this phase, chemotherapy dose reductions to 75% of the planned dose were required in one patient receiving VIPD because of grade 2 nephrotoxicity and in two patients treated with GELOX/PGEMOX owing to poor treatment tolerance and grade 4 neutropenia.

### 3.3. Treatment Response

In the concurrent chemoradiotherapy (CCRT) cohorts receiving adjuvant VIPD or VIDL, all patients successfully completed the concurrent treatment phase. The complete response (CR) rate increased substantially from 55.6 to 68.8% following CCRT to 81.5–93.8% at the completion of the entire treatment course. Similarly, in the sequential GELOX/PGEMOX-based cohort, the CR rate improved progressively from 10.5% after induction chemotherapy to 63.2% following radiotherapy, ultimately reaching 84.2% after completion of consolidation chemotherapy. These findings underscore the pivotal role of systemic adjuvant chemotherapy in consolidating initial treatment responses and minimizing the risk of disease progression.

Among the three treatment strategies, Arm 2 achieved the highest complete response rate and no cases of progressive disease; however, the difference did not reach statistical significance (*p* = 0.458). Detailed treatment response outcomes are summarized in Table 2.

The analysis demonstrated that patients with undetectable or low baseline plasma EBV-DNA levels (<1000 copies/mL) achieved an objective response in nearly all cases, with the vast majority attaining either complete or partial remission. In contrast, 20.0% of patients with high EBV-DNA levels (≥10^3^ copies/mL) failed to achieve a treatment response. This association was statistically significant (*p* = 0.041). On univariate analysis, no other clinicopathologic variables, including sex, B symptoms, primary tumor location, serum lactate dehydrogenase (LDH), beta-2 microglobulin level, disease stage, or PINKE score, demonstrated a statistically significant association with treatment response. Detailed response rates according to selected clinical and laboratory variables are presented in Table 3.

### 3.4. Survival

**Figure 1:**
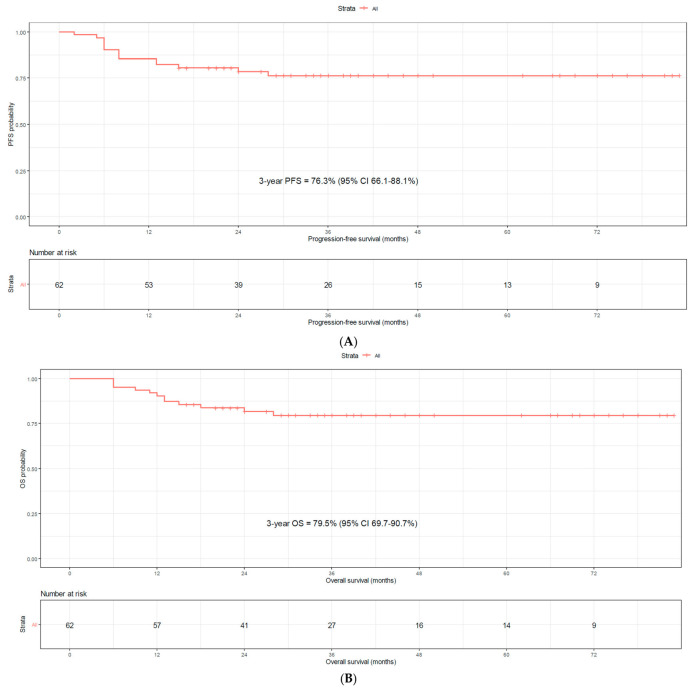
Progression-free survival (PFS) (**A**) and overall survival (OS) (**B**).

The median follow-up duration for the entire cohort was 33 months (range, 6–83 months). Median follow-up was 62 months in Arm 1, 38 months in Arm 2, and 21 months in Arm 3, reflecting the sequential implementation of different treatment strategies over the study period. At the time of analysis, 50 of the 62 patients remained alive, corresponding to an overall survival proportion of 79.5% (95%CI 69.7–90.7%). Kaplan–Meier analysis demonstrated an estimated 3-year progression-free survival (PFS) rate of 76.3% (95%CI 66.1–88.1%) (Figure 1). The shortest recorded PFS duration was 2 months, whereas the longest follow-up extended to 83 months. Most progression events occurred within the first 30 months following treatment initiation, after which the PFS curve plateaued and remained relatively stable. Median PFS and overall survival (OS) were not reached during the study period.

**Figure 2:**
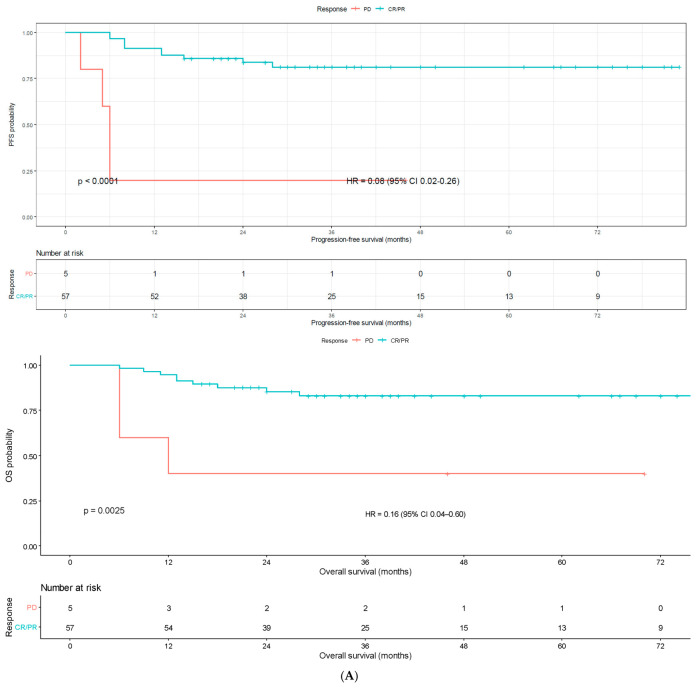

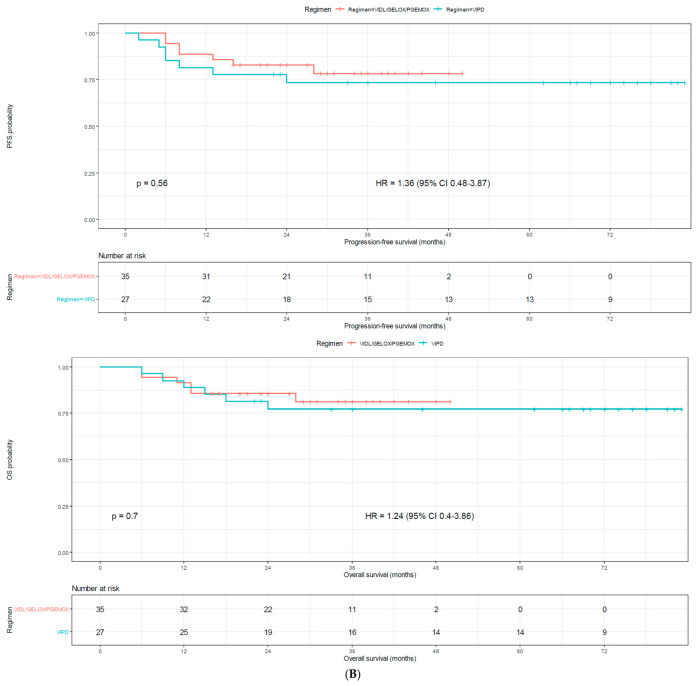

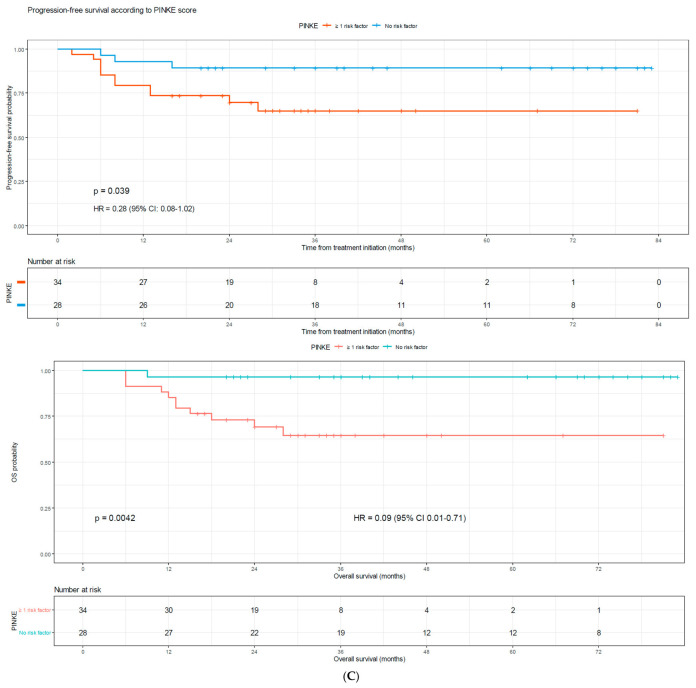
Kaplan–Meier survival curves according to (**A**) treatment response, (**B**) treatment regimen, and (**C**) PINKE risk stratification.

The estimated 3-year Kaplan–Meier OS rate was 79.5% (95%CI 69.7–90.7%). No additional deaths were observed beyond 28 months of follow-up, and the survival curve subsequently remained stable through the end of the observation period. Patients who achieved complete response (CR) exhibited significantly superior 3-year PFS and OS compared with those who failed to achieve CR. The highly significant *p*-value (<0.001) underscores the substantial prognostic impact of first-line treatment efficacy on long-term survival outcomes. Kaplan–Meier estimates of PFS and OS according to treatment response, treatment regimens, and PINKE risk factors are presented in Figure 2A–C. Multivariate Cox regression analysis demonstrated that patients without PINKE risk factors experienced significantly superior overall survival (OS) and progression-free survival (PFS) compared with those presenting with at least one PINKE risk factor (Table 4). The presence of ≥1 PINKE risk factor was associated with inferior survival outcomes, with statistically significant differences observed for both OS (*p* = 0.009) and PFS (*p* = 0.006).

### 3.5. Toxicity

#### Adverse Events in Radiotherapy Phase (With or Without Chemotherapy)

With respect to hematologic toxicity, anemia, neutropenia, and thrombocytopenia were observed in 37.1%, 17.7%, and 17.7% of patients, respectively. The majority of these adverse events were mild to moderate in severity (grade 1–2). Grade 3 neutropenia occurred in 3.2% of patients, whereas grade 3 thrombocytopenia was documented in 4.8%; importantly, both events were clinically asymptomatic. No grade 4 hematologic toxicities were recorded during the study period.

The most commonly reported non-hematologic adverse events during the chemoradiotherapy phase were elevated hepatic transaminases (30.6%), nausea (30.6%), and vomiting (29.0%), most of which were classified as grade 1–2 toxicities. Severe non-hematologic toxicities were uncommon, with grade 4 elevations in serum lipase and electrolyte disturbances each occurring in 1.6% of patients.

Regarding radiotherapy-related adverse events, radiation dermatitis represented the most frequent toxicity, occurring in 82.2% of patients; however, the overwhelming majority of cases were limited to grade 1 severity (80.6%). Radiation-induced mucositis was also frequently observed, affecting 64.5% of patients, predominantly as grade 1 events. Xerostomia was reported in 14.5% of cases. Notably, no grade 3 or grade 4 radiation-associated toxicities were identified throughout the study period (Table 5).

### 3.6. Adverse Events in Chemotherapy Alone Phase

During the adjuvant chemotherapy phase, anemia represented the most frequently observed hematologic toxicity, occurring in 57.7% of patients. Most cases were low grade, comprising grade 1 anemia in 37.3% and grade 2 anemia in 13.6% of patients, whereas grade 3 anemia was documented in 6.8% of cases. The overall incidence of neutropenia was 32.2%, including grade 3 and grade 4 events in 6.8% and 11.9% of patients, respectively. Thrombocytopenia occurred less frequently, affecting 18.6% of the cohort, and was predominantly limited to grade 1 severity, with no reported grade 3 or grade 4 thrombocytopenic events (Table 5).

With regard to non-hematologic toxicities, elevated hepatic transaminases constituted the most common adverse event, occurring in 28.8% of patients. Gastrointestinal toxicities were also frequently observed, with nausea and vomiting reported in 25.5% and 23.8% of patients, respectively. The majority of non-hematologic adverse events were mild to moderate in severity (grade 1–2). Nevertheless, a limited number of grade 3 toxicities were documented, including diarrhea, hyperlipasemia, hyperamylasemia, and hypofibrinogenemia. Grade 1 infusion-related reactions occurred in seven patients (11.9%). Notably, one patient developed a grade 4 hypersensitivity reaction to L-asparaginase, necessitating treatment discontinuation and subsequent transition to the VIPD regimen (Table 5).

## 4. Discussion

The baseline characteristics of the 62-patient cohort at K Hospital consistently reflect the epidemiological features of extranodal natural killer/T-cell lymphoma (ENKTL) observed across Southeast and East Asia. The mean age of 44.3 ± 12.0 years and the significant male predominance (75.8%) align with regional data, such as studies from South Korea where the median age is approximately 50 years [12,13]. This demographic profile, characterized by disease onset during the prime working years, necessitates therapeutic strategies that balance high efficacy with manageable long-term toxicity to preserve quality of life post-treatment. Furthermore, the majority of patients presented with stage IE disease (67.7%) and good performance status (ECOG PS 0 in 87.1%), reflecting current diagnostic trends that allow for intervention at localized stages where curative intent is most achievable [14]. Baseline characteristics differed across the three treatment groups. Compared with the VIPD and VIDL groups, patients treated with GELOX/PGEMOX more often presented with stage IIE disease, higher baseline EBV-DNA levels, and higher PINKE scores. These differences are consistent with treatment selection in routine clinical practice and may have contributed to the observed treatment outcomes. As a result, comparisons between treatment strategies should be interpreted cautiously.

A central finding of this study is the marked efficacy of non-anthracycline-based multimodal regimens. The poor efficacy of anthracycline-containing regimens in ENKTL has been attributed to intrinsic multidrug resistance mechanisms, particularly overexpression of efflux transport proteins, thereby supporting the transition toward platinum- and asparaginase-based approaches [15]. By shifting to agents unaffected by this mechanism—specifically cisplatin and asparaginase—we observed complete response (CR) rates of 81.5–93.8% for the CCRT followed by VIPD/VIDL cohort and 84.2% for the sequential GELOX/PGEMOX cohort. These results are highly comparable to international benchmarks, such as the 80.0% CR rate reported in the Kim et al. VIPD trial and the 87% rate in the VIDL phase II study [12,13]. The significant increase in CR from 60.5% post-CCRT to 81.5–93.8% at the end of treatment in our study underscores the critical role of systemic adjuvant chemotherapy in eradicating micrometastases and consolidating initial local responses [12,13].

Survival outcomes further validate the strength of these combined modalities. The estimated 3-year overall survival (OS) of 79.5% and progression-free survival (PFS) of 76.3% in our cohort fall within the range of outcomes reported in major Chinese and Korean studies. Notably, the progression events primarily occurred within the first 30 months, after which the PFS curve stabilized, suggesting that patients who achieve durable remission for two to three years have a high probability of long-term cure. Patients achieving complete response exhibited significantly superior PFS and OS on Kaplan–Meier analysis, highlighting the prognostic importance of treatment efficacy. This observation is consistent with findings from the Beijing Tongren Hospital study of 266 patients, in which immediate post-treatment CR was identified as the sole independent prognostic factor for both PFS and OS [16]. Moreover, multivariable Cox regression analysis in our cohort identified the PINKE prognostic model as a significant determinant of survival outcomes. Patients without PINKE risk factors demonstrated significantly superior OS and PFS compared with those harboring at least one adverse PINKE factor. The presence of ≥1 PINKE risk factor was associated with inferior survival, with statistically significant differences observed for both PFS (*p* = 0.006) and OS (*p* = 0.009). These findings further validate the prognostic utility of the PINKE model for risk stratification and support its use in identifying patients who may benefit from intensified therapeutic strategies or closer post-treatment surveillance. When interpreting these survival outcomes, the different follow-up durations across treatment groups should also be considered. Patients in Arms 1, 2, and 3 had median follow-up durations of 62, 38, and 21 months, respectively, reflecting the sequential implementation of evolving treatment protocols over the study period. These changes were driven by improvements in drug availability and the evolution of routine clinical practice in Vietnam. Consequently, direct comparisons of progression-free and overall survival between treatment strategies should be interpreted with caution, as differences in follow-up duration may have introduced temporal bias.

The role of EBV-DNA as a molecular biomarker was evident in its strong clinical correlation with treatment response. In this cohort, a negative or low pre-treatment viral load (<1000 copies/mL) was associated with a 97.9% response rate, suggesting that low viral burden may serve as a favorable indicator for chemosensitivity. Conversely, patients with high viral loads (≥1000 copies/mL) demonstrated a higher incidence of treatment failure (20.0%). This association reached statistical significance (*p* = 0.041), supporting the potential role of baseline EBV-DNA as a clinically relevant biomarker of treatment response. The observed trend aligns with larger studies suggesting that circulating EBV-DNA reflects tumor burden and remains a vital tool for risk stratification and monitoring in clinical practice [17].

Safety and toxicity management remain significant challenges when implementing these intensive protocols. While concurrent chemoradiotherapy with weekly cisplatin was well-tolerated with predominantly grade 1–2 toxicities, the systemic chemotherapy phases introduced more severe complications. Grade 4 neutropenia occurred in 11.9% of our patients during the chemotherapy-only phase, necessitating vigilant monitoring and the use of growth factors. Asparaginase-specific toxicities, including hypofibrinogenemia (17.0%) and liver dysfunction (28.8%), were also prevalent but manageable. The occurrence of one grade 4 hypersensitivity reaction to L-asparaginase highlights the clinical utility of pegaspargase, which offers a longer half-life and lower immunogenicity, as utilized in our PGEMOX subgroup. Overall, the frequency of infusion-related reactions and high-grade adverse effects (grade 3–4) was acceptable, showing no significant deviation from data reported in studies using different regimens [13,17,18].

Radiotherapy (RT) plays a central role in the treatment of localized extranodal NK/T-cell lymphoma (ENKTL), nasal type. It may be used alone or combined with chemotherapy, either concurrently or sequentially. Current guidelines recommend involved-site radiotherapy (ISRT), which limits the radiation field to sites of known disease [19]. The clinical target volume (CTV) includes the gross tumor volume (GTV) identified on CT or MRI, together with the ipsilateral nasal cavity and adjacent structures, including the contralateral nasal cavity, maxillary sinus, ethmoid sinuses, and nasopharynx.

For sequential radiotherapy patients, the recommended total dose ranges from 50 to 55 Gy. This is supported by a study of 74 patients with localized ENKTL, which demonstrated that a dose of ≥54 Gy significantly improved 5-year overall survival (OS) and disease-free survival (DFS) rates (76% and 60%, respectively) compared to those receiving <54 Gy (46% and 33%) [20]. In concurrent chemoradiotherapy settings, the prescribed dose typically varies between 40 and 56 Gy, depending on the specific regimen and integration method [13,14,21,22]. Notably, the definition of target volumes remains consistent with monotherapy; even in cases where induction chemotherapy achieves a complete response, the CTV must include the initial pre-chemotherapy tumor extent.

Future studies should focus on validating these findings in larger multicenter cohorts and exploring novel therapeutic approaches for high-risk patients. Given the biological association between ENKTL and Epstein–Barr virus infection, immune checkpoint inhibitors and cellular therapies have emerged as promising treatment strategies in relapsed or refractory disease. However, their role in the frontline management of localized ENKTL remains to be established through prospective clinical studies [23,24].

Several limitations of the present study should be acknowledged. First, as this was a single-center study conducted at K Hospital, the findings may reflect institution-specific treatment practices and therefore may not be entirely generalizable to other healthcare settings or populations. Second, incomplete data regarding Epstein–Barr virus-encoded RNA in situ hybridization (EBER-ISH) represented an additional limitation of the present study. Only 32.3% of patients underwent EBER-ISH testing, although all tested cases demonstrated positive results. This limitation primarily reflected real-world economic constraints, as EBER-ISH was not routinely reimbursed and some patients were either unable or unwilling to undergo additional out-of-pocket investigations. Furthermore, direct comparisons between the three treatment strategies should be interpreted with caution. The treatment protocols were introduced sequentially between 2019 and 2025, reflecting changes in drug availability and routine clinical practice in Vietnam. Consequently, follow-up duration differed across the treatment groups, and patients treated with GELOX/PGEMOX generally had a less favorable baseline profile, including more advanced disease, higher EBV-DNA levels, and higher PINKE scores than those receiving VIPD or VIDL. Improvements in supportive care, diagnostic imaging, and accumulated clinical experience over the study period may also have contributed to the observed outcomes. Given the limited sample size and the small number of outcome events, we did not perform additional multivariable or sensitivity analyses. Therefore, residual confounding and temporal bias cannot be excluded when interpreting the differences between treatment strategies. Finally, our study focused primarily on acute treatment-related toxicities. Although previous studies have described late radiotherapy-related complications, particularly xerostomia and toxicities involving adjacent head and neck structures, no clinically significant late toxicities were identified during follow-up in our cohort. Nevertheless, the retrospective design and the relatively shorter follow-up of patients treated with the most recent regimen may have limited the detection of late adverse events.

## 5. Conclusions

In conclusion, non-anthracycline-based regimens combined with contemporary radiotherapy achieved high response rates and demonstrated acceptable safety profiles in patients with localized extranodal NK/T-cell lymphoma. Favorable clinical outcomes were also observed in selected patients treated with non-asparaginase-containing regimens when combined with intensive radiotherapy. Although asparaginase-containing regimens were associated with specific toxicities, including infusion-related reactions and elevations in pancreatic enzymes, most adverse events were limited to grade 1–2 severity and were clinically manageable.

Importantly, the PINKE prognostic model remained an independent prognostic factor for both progression-free survival (PFS) and overall survival (OS). Collectively, our findings support the feasibility and effectiveness of multimodal treatment strategies in real-world clinical practice and highlight the importance of individualized therapeutic decision-making based on patient tolerance, toxicity risk, and resource availability in resource-constrained healthcare settings.

## Figures and Tables

**Table 1:** Baseline clinicopathological characteristics of the study population.

		Whole Cohort (*n* = 62)	VIPD Arm 1 (*n* = 27)	VIDL Arm 2 (*n* = 16)	GELOX /PGEMOX Arm 3 (*n* = 19)	*p*
Age median (range)		44 (17–71)	46 (21–71)	45 (17–66)	42 (29–66)	0.68 ^@^
Age group n (%)	<30	9 (14.5)	4 (14.8)	4 (25)	1 (5.3)	0.245 *
	30–49	32 (51.6)	11 (40.7)	7 (43.8)	14 (73.7)	
	50–69	20 (32.3)	11 (40.7)	5 (31.2)	4 (21.1)	
	≥70	1 (1.6)	1 (3.7)	0	0	
Male n (%)		47 (75.8)	20 (74.1)	14 (87.5)	13 (68.4)	0.45 *
ECOG PS n (%)	0	54 (87.1)	25 (92.6)	13 (81.4)	16 (84.2)	0.64 *
	1	6 (9.1)	2 (7.4)	2 (1.3)	2 (10.5)	
	2	2 (3.2)	0	1 (6.3)	1 (5.3)	
B syndrome n (%)	Yes	27 (43.5)	10 (37)	9 (56.2)	8 (42.1)	0.49 *
	No	35 (56.5)	17 (63)	7 (43.8)	11 (57.9)	
Primary site n (%)	Nasal cavity	43 (69.4)	20 (74.1)	12 (75)	11 (57.9)	0.22 *
	Sinuses	4 (6.5)	1 (3.7)	2 (12.5)	1 (5.3)	
	Nasopharynx	8 (12.9)	5 (18.5)	1 (6.2)	2 (10.5)	
	Palate	2 (3.2)	1 (3.7)	0	1 (5.3)	
	Extensive involvement of the nasal–paranasal region	5 (8.1)	0	1 (6.2)	4 (21.1)	
EBV-DNA (copies/mL) n (%)	Negative	33 (53.2)	17 (63.0)	10 (62.5)	6 (31.6)	0.026 *
	<1000	14 (22.6)	7 (25.9)	4 (25.0)	3 (15.8)	
	≥1000	15 (24.2)	3 (11.1)	2 (12.5)	10 (52.6)	
Stage n (%)	IE	42 (67.7)	23 (85.2)	11 (68.8)	8 (42.1)	0.008 *
	IIE	20 (32.3)	4 (14.8)	5 (31.2)	11 (57.9)	
PINKE score n (%)	0	28 (45.2)	19 (70.4)	6 (37.5)	3 (15.8)	0.001 *
	1	27 (43.5)	8 (29.6)	8 (50.0)	11 (57.9)	
	2	7 (11.3)	0	2 (12.5)	5 (26.3)	
LDH concentration n (%)	Normal	47 (75.8)	23 (85.2)	12 (75.0)	12 (63.2)	0.212 *
	Elevated	15 (24.2)	4 (14.8)	4 (25.0)	7 (36.8)	
β2-microglobulin n (%)	Normal	30 (48.4)	12 (44.4)	6 (37.5)	12 (63.2)	0.291 *
	Elevated	32 (51.6)	15 (55.6)	10 (62.5)	7 (36.8)	

^@^ Kruskal–Wallis test. * Fisher’s exact test. Abbreviations: ECOG, Eastern Cooperative Oncology Group; LDH, lactate dehydrogenase; EBV, Epstein–Barr virus; PINKE, Prognostic Index of Natural Killer lymphoma incorporating EBV-DNA.

**Table 2:** Treatment response according to treatment phase and treatment strategy.

Response	VIPD Arm 1 (*n* = 27)		VIDL Arm 2 (*n* = 16)		GELOX /PGEMOX Arm 3 (*n* = 19)		
	After CCRT—*n* (%)	End of First Line Treatment *n* (%)	After CCRT—*n* (%)	End of First Line Treatment *n* (%)	After Induction CT—*n* (%)	After RT *n* (%)	End of First Line Treatment *n* (%)
CR	15 (55.6%)	22 (81.5%)	11 (68.8%)	15 (93.8%)	2 (10.5%)	12 (63.2%)	16 (84.2%)
PR	11 (40.7%)	2 (7.4%)	4 (25%)	1 (6.2%)	16 (84.2%)	6 (31.6%)	2 (10.5%)
SD	0	0	1 (6.2%)	0	0 (0)	0 (0)	0 (0.0)
PD	1 (3.7%)	3 (11.1%)	0	0	1 (5.3%)	1 (5.3%)	1 (5.3%)
Total	27	27 *	16	16	19	19	19

* two patients in Arm 1 did not advance to adjuvant chemotherapy, one due to disease progression, while one with partial response declined further treatment for personal reasons. Both patients were retained in the final response analysis, and their last documented response was used as the end-of-treatment assessment. Abbreviations: CCRT, concurrent chemoradiotherapy; VIPD, etoposide/ifosfamide/cisplatin/dexamethasone; VIDL, etoposide/ifosfamide/dexamethasone/L-asparaginase.

**Table 3:** Prognostic factors associated with treatment response by univariate analysis.

Variable	Response *n* (%)	Non-Response *n* (%)	Total *n*	*p* Univariate
Age group				
≤60	53 (93%)	4 (7%)	57	1.000 *
>60	5 (100%)	0	5	
Gender				
Male	44 (93.6)	3 (6.4)	47	1.000 *
Female	14 (93.3)	1 (6.7)	15	
ECOG				
0	50 (92.6)	4 (7.4)	54	1.000 *
1	6 (100)	0	6	
2	2 (100)	0	2	
B symptoms				
Yes	25 (92.6)	2 (7.4)	27	1.000 *
No	33 (94.3)	2 (5.7)	35	
Tumor location				
Nasal	40 (93.0)	3 (7.0)	43	1.000 *
Others	18 (94.7)	1 (5.3)	19	
LDH				
Elevated	14 (93.3)	1 (6.7)	15	1.000 *
Normal	44 (93.6)	3 (6.4)	47	
β2-microglobulin				
Elevated	31 (96.9)	1 (3.1)	32	0.346 *
Normal	27 (90.0)	3 (10.0)	30	
Stage				
IE	40 (95.2)	2 (4.8)	42	0.588 *
IIE	18 (90.0)	2 (10.0)	20	
PINKE score				
0	27 (96.4)	1 (3.6)	28	0.620 *
1–2	31 (91.2)	3 (8.8)	34	
EBV-DNA (copies/mL)				
Negative or <1000	46 (97.9)	1 (2.1)	47	0.041 *
≥1000	12 (80.0)	3 (20.0)	15	
Regimens				
CCRT + VIPD	24 (88.9)	3 (11.1)	27	0.458 *
CCRT + VIDL	16 (100)	0	16	
GELOX/PGEMOX + RT	18 (94.7)	1 (5.3)	19	

* Fisher’s exact test. Response was defined as complete response or partial response.

**Table 4:** Multivariable Cox regression analysis for progression-free survival (PFS) and overall survival (OS).

Prognostic Factor *	PFS HR	95% CI	*p* Value	OS HR	95% CI	*p* Value
ECOG PS (0 vs. ≥1)	0.717	0.150–3.416	0.676	0.724	0.153–3.431	0.685
LDH (normal vs. ≥ULN)	1.231	0.300–5.055	0.773	1.608	0.362–7.142	0.532
β2-microglobulin (normal vs. ≥ULN)	0.707	0.200–2.508	0.592	0.865	0.233–3.215	0.829
B symptoms (no vs. yes)	1.162	0.327–4.129	0.817	1.154	0.328–4.053	0.823
PINKE (no risk factor vs. ≥1 risk factor)	0.136	0.031–0.603	0.009	0.046	0.005–0.409	0.006
Regimen (VIPD vs. VIDL/GELOX)	0.285	0.078–1.035	0.056	0.260	0.067–1.008	0.051

Abbreviations: HR, hazard ratio; CI, confidence interval; ECOG PS, Eastern Cooperative Oncology Group performance status; LDH, lactate dehydrogenase; ULN, upper limit of normal. * variables were selected based on clinical relevance and entered into multivariable Cox proportional hazards regression models for PFS and OS. *p*-values derived from multivariate Cox proportional hazards models.

**Table 5:** Treatment-related adverse events according to treatment phase.

Adverse Event	Radiotherapy/CCRT Phase Grade 1–2 *n* (%)	Radiotherapy/CCRT Phase Grade 3–4 *n* (%)	Overall *n* (%)	Chemotherapy Phase Grade 1–2 *n* (%)	Chemotherapy Phase Grade 3–4 *n* (%)	Overall *n* (%)
**Hematological**						
Anemia	22 (35.5)	1 (1.6)	23 (37.1)	30 (50.9)	4 (6.8)	34 (57.7)
Neutropenia	9 (14.5)	2 (3.2)	11 (17.7)	8 (13.6)	11 (18.7)	19 (32.3)
Thrombocytopenia	8 (12.9)	3 (4.8)	11 (17.7)	11 (18.6)	0 (0.0)	11 (18.6)
**Biochemical**						
Elevated ALT/AST	17 (27.4)	2 (3.2)	19 (30.6)	17 (28.8)	0 (0.0)	17 (28.8)
Renal impairment	2 (3.2)	0 (0.0)	2 (3.2)	3 (5.1)	0 (0.0)	3 (5.1)
Elevated lipase	7 (11.3)	3 (4.8)	10 (16.1)	8 (13.6)	2 (3.4)	10 (17.0)
Elevated amylase	0 (0.0)	0 (0.0)	0 (0.0)	0 (0.0)	1 (1.7)	1 (1.7)
Electrolyte imbalance	6 (9.7)	2 (3.2)	8 (12.9)	3 (5.1)	0 (0.0)	3 (5.1)
Hypofibrinogenemia	4 (6.5)	1 (1.6)	5 (8.1)	9 (15.3)	1 (1.7)	10 (17.0)
**Gastrointestinal**						
Nausea	19 (30.6)	0 (0.0)	19 (30.6)	15 (25.5)	0 (0.0)	15 (25.5)
Vomiting	18 (29.0)	0 (0.0)	18 (29.0)	14 (23.8)	0 (0.0)	14 (23.8)
Diarrhea	4 (6.5)	2 (3.2)	6 (9.7)	2 (3.4)	1 (1.7)	3 (5.1)
Anorexia	—	—	—	6 (10.2)	0 (0.0)	6 (10.2)
**Others**						
Radiation dermatitis	51 (82.2)	0 (0.0)	51 (82.2)	—	—	—
Radiation mucositis	40 (64.5)	0 (0.0)	40 (64.5)	—	—	—
Xerostomia	9 (14.5)	0 (0.0)	9 (14.5)	—	—	—
Peripheral neuropathy	—	—	—	5 (8.5)	0 (0.0)	5 (8.5)
Alopecia	—	—	—	5 (8.5)	0 (0.0)	5 (8.5)
Infusion reaction	2 (3.2)	0 (0.0)	2 (3.2)	7 (11.9)	1 (1.7)	8 (13.6)
Thrombosis	0 (0.0)	0 (0.0)	0 (0.0)	0 (0.0)	0 (0.0)	0 (0.0)

Adverse events were graded according to the Common Terminology Criteria for Adverse Events (CTCAE), version 5.0. Abbreviations: CCRT, concurrent chemoradiotherapy; ALT, alanine aminotransferase; AST, aspartate aminotransferase.

## Data Availability

The datasets generated and/or analyzed during the current study are not publicly available due to patient privacy and ethical restrictions but are available from the corresponding author on reasonable request and with permission from the Institutional Review Board.

## Supplementary Materials

The following supporting information can be downloaded at: https://www.mdpi.com/article/10.3390/curroncol33070435/s1, File S1: STROBE Checklist for Cohort Studies.

## Abbreviations

The following abbreviations are used in this manuscript:

ALT	Alanine aminotransferase
AST	Aspartate aminotransferase
APTT	Activated partial thromboplastin time
CCRT	Concurrent chemoradiotherapy
CI	Confidence interval
CR	Complete response
CT	Computed tomography
CTCAE	Common Terminology Criteria for Adverse Events
CTV	Clinical target volume
EBER-ISH	Epstein–Barr virus-encoded RNA in situ hybridization
EBV	Epstein–Barr virus
ECOG	Eastern Cooperative Oncology Group
ENKTL-NT	Extranodal natural killer/T-cell lymphoma, nasal type
FDG	Fluorodeoxyglucose
GELOX	Gemcitabine, oxaliplatin, and L-asparaginase
GTV	Gross tumor volume
HR	Hazard ratio
IMRT	Intensity-modulated radiotherapy
INR	International normalized ratio
ISRT	Involved-site radiotherapy
LDH	Lactate dehydrogenase
MRI	Magnetic resonance imaging
NCCN	National Comprehensive Cancer Network
NK	Natural killer
OS	Overall survival
PCR	Polymerase chain reaction
PD	Progressive disease
PET/CT	Positron emission tomography/computed tomography
PFS	Progression-free survival
PGEMOX	Pegaspargase, gemcitabine, and oxaliplatin
PINKE	Prognostic Index of Natural Killer Lymphoma Incorporating Epstein–Barr Virus DNA
PR	Partial response
PT	Prothrombin time
RT	Radiotherapy
SD	Stable disease
ULN	Upper limit of normal
VIDL	Etoposide, ifosfamide, dexamethasone, and L-asparaginase
VIPD	Etoposide, ifosfamide, cisplatin, and dexamethasone
VMAT	Volumetric modulated arc therapy
WHO	World Health Organization
