# Supplementary material for: Real-World Outcomes of Frontline Multimodal Treatment Strategies for Localized Extranodal NK/T-Cell Lymphoma, Nasal Type: A Single-Center Vietnamese Cohort Study
Source: Curr Oncol. 2026 Jul 21;33(7):435. doi: 10.3390/curroncol33070435 (PMC13409056; doi:10.3390/curroncol33070435)
Supplement: Supplementary file 1 [file curroncol-33-00435-s001.zip › curroncol-4405529-supplementary.pdf]

STROBE Checklist for Cohort Studies

Manuscript title: Real-World Outcomes of Frontline Multimodal Treatment Strategies for Localized Extranodal NK/T-Cell Lymphoma, Nasal Type: A Single-Center Vietnamese Cohort Study

Section/Item	Item No.	Recommendation	Updated Page No.
Title and abstract	1a	Indicate the study's design with a commonly used term in the title or abstract.	1–2
Title and abstract	1b	Provide an informative and balanced summary.	1–2
Introduction	2	Explain the scientific background and rationale.	2–3
Introduction	3	State specific objectives.	3
Methods	4	Present key elements of study design.	3–4
Methods	5	Describe the setting, locations and dates.	3–4
Methods	6a	Give eligibility criteria and participant selection.	3
Methods	6b	Matching criteria (if applicable).	N/A
Methods	7	Clearly define outcomes, exposures, predictors and confounders.	4–6
Methods	8	Data sources and measurements.	5–6
Methods	9	Describe efforts to address potential bias.	4
Methods	10	Explain how study size was arrived at.	3, 6
Methods	11	Handling of quantitative variables.	6
Methods	12a	Statistical methods.	6
Methods	12b	Methods for subgroup analyses.	10–13
Methods	12c	Explain how missing data were addressed.	6
Methods	12d	Explain loss to follow-up.	3–4
Methods	12e	Sensitivity analyses.	N/A
Results	13a	Numbers of individuals at each stage.	6–9
Results	13b	Reasons for non-participation.	3, 8
Results	13c	Flow diagram.	N/A
Results	14a	Participant characteristics.	6–7
Results	14b	Missing data.	6
Results	14c	Summarise follow-up time.	9–10
Results	15	Outcome data.	8–15
Results	16	Main results (estimates and precision).	9–13
Results	17	Other analyses.	10–13
Discussion	18	Summarise key results.	15–18
Discussion	19	Discuss limitations.	18–20
Discussion	20	Provide cautious interpretation.	15–20
Discussion	21	Discuss generalisability.	19–20
Other information	22	Funding.	20–21
